# Conservative Management of Paediatric Clavicle Fractures

**DOI:** 10.1155/2011/172571

**Published:** 2011-12-06

**Authors:** Barry J. O'Neill, Alan P. Molloy, William Curtin

**Affiliations:** Department of Orthopaedics & Trauma, Galway Regional Hospitals, Galway, Ireland

## Abstract

Paediatric clavicle fractures have traditionally been treated nonoperatively. Recent studies have recommended operative management for displaced midshaft fractures. We conducted a retrospective review of all clavicle fractures in children aged one to sixteen over a two-year period. We classified fractures and evaluated followup and clinical outcome. We identified 190 fractures. There were 135 boys and 55 girls. 65% of fractures were displaced and 35% undisplaced. Mean radiographic and clinical followup was 35 days and 44 days, respectively. Clavicle fractures in children heal with nonoperative management. Radiographs of clavicle fractures in children are unnecessary in the absence of clinical symptoms.

## 1. Introduction

Clavicle fractures occur frequently, with the reported rates ranging between 8% and 15% of all paediatric fractures [1–3]. The vast majority of these injuries can be treated nonoperatively with excellent results [4, 5]. Reported indications for operative management include markedly displaced fractures with compromised skin integrity, open fractures, concomitant vascular injury requiring repair, and compromise of the brachial plexus [6–10]. These reports, however, have mostly referred to fractures within the adult population. More recently, there has been some support for operative management of middle third fractures with marked displacement or shortening [11–13]. Some of these studies have specifically recommended fixation in children and adolescents [14, 15]. The aim of this study was to review the outcome of clavicle fractures in paediatric patients at our institute and to determine the number of such fractures that require operative management.

## 2. Methods

We retrospectively reviewed all clavicle fractures in children treated at our institute over a two-year period. We used the AGFA IMPAX Web1000 system to identify all radiographs of the shoulder region performed in children aged up to and including 15 years old. These radiographs were then reviewed to identify all clavicle fractures in the patient cohort group. Medical records and theatre records were then reviewed to establish the classification of each fracture, the treatment method used, the duration of radiographic followup, the duration of clinical followup, and the clinical outcome. Exclusion criteria were any patient aged 16 years or older, and any fracture as a result of birth trauma.

## 3. Results

**Figure 1:**
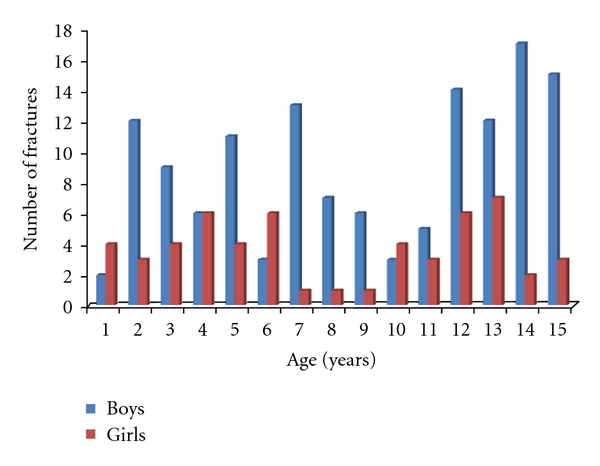
Age and sex distribution of fractures.

**Figure 2:**
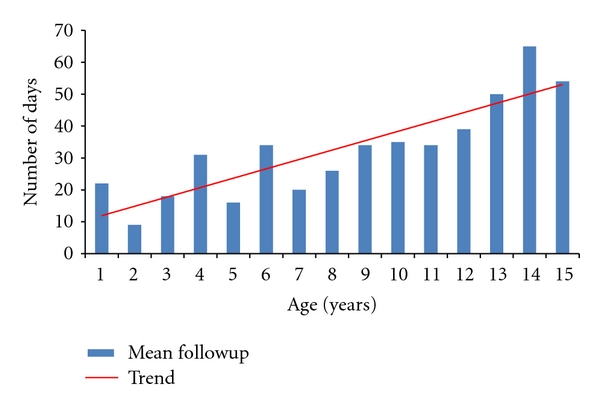
Mean radiological followup fractures by age, expressed in days.

**Figure 3:**
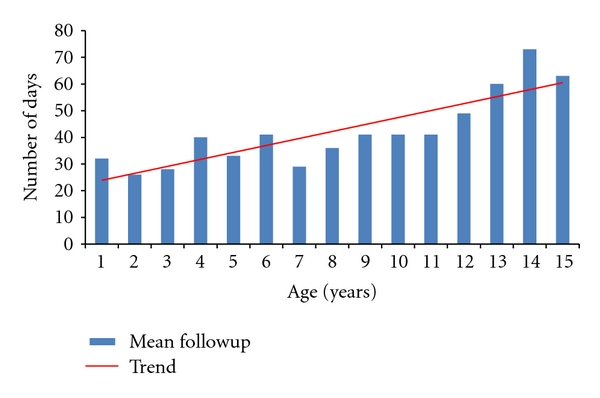
Mean clinical followup fractures by age expressed in days.

We identified 487 clavicle fractures in 483 patients treated in our institute during the two-year period. Of these, 190 clavicle fractures satisfied the inclusion criteria (39%). Ten neonates were excluded because their injuries were related to birth trauma, and 283 patients were excluded because they were 16 years old or older. There were 135 fractures in boys and 55 fractures in girls. The age and sex breakdown of all fractures can be seen in Figure 1. All fractures were classified using the system described by Robinson [16, Table  1]. The classification of all 190 fractures can be seen in Table 1. One hundred and twenty-four fractures were undisplaced (65%) and 66 were displaced (35%). All fractures were treated with analgesia and a simple broad-arm sling. Immobilisation in the sling was continued until the patient was comfortable enough to mobilise without support. Radiographs were taken at first presentation. Further radiographs were only taken if the patient continued to have pain or limitation of function when reviewed. Initial review was within one week of injury, and second review was at three weeks after initial review. Further review appointments were arranged at the discretion of the clinician and were determined by clinical and/or radiographic assessment. The mean radiographic follow-up of the group as a whole was 35 days (5 weeks), and the mean clinical follow-up was 44 days (6.3 weeks). The mean radiological and clinical follow-up of the group when subdivided by age can be seen in Figures 2 and 3. Forty-four of 190 fractures (23%) had radiographic confirmation of fracture healing. The remaining 77% had radiographic examinations discontinued when clinical symptoms of pain and limitation of function had resolved. All fractures in this study had healed clinically when the child was discharged from follow-up. All fractures healed clinically with nonoperative management, and no children required surgical intervention.

## 4. Discussion

Clavicle fractures are common injuries in general, and, in this study, 39% of all fractures treated over a two-year period involved children of 15 years or younger. Despite this, there are surprisingly few published studies that specifically discuss paediatric clavicle fractures. Traditionally, clavicle fractures have been treated nonoperatively, particularly in children. This is largely due to the relatively low incidence of complications following non-operative management. Indications for operative management in the acute setting have included markedly displaced fractures with compromised skin integrity, open fractures, concomitant vascular injury requiring repair, and compromise of the brachial plexus [6–10]. Most of these studies, however, describe these complications occurring in adults. Howard and Shafer described fourteen clavicle fractures with associated neurovascular complications, but only one case occurred in a child [6], a ten-year old with a depressed clavicle fracture compressing the subclavian vein. Mital and Aufranc also described a venous occlusion following a greenstick fracture of the clavicle [17]. Keating and Von Ungern-Sternberg recently published a case report entitled “Compression of the common carotid artery following clavicle fracture in a twelve-year-old” but the report actually describes a clavicle dislocation [18]. Fixation of a clavicle fracture associated with a dislocation [19, 20] and fixation of a fracture associated with a sternoclavicular physeal fracture [21] have also been described in children. Operative management for nonunion of a clavicle fracture in a child has also been described [22].

Reports of complications of clavicle fractures and operative management of clavicle fractures in paediatric patients are few. The examples cited above demonstrate that complications do occur, but these are extremely rare. In our group of 190 patients, none had significant associated injuries and none required operative management.

**Figure 4:**
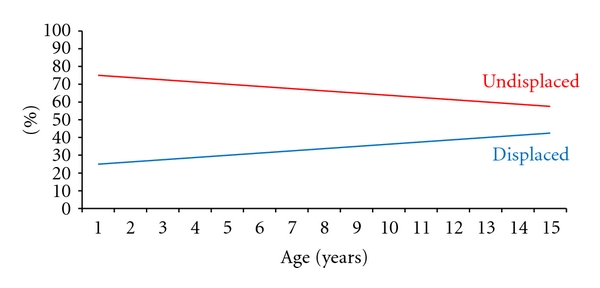
Trends of displaced and undisplaced fractures expressed as a percentage of total for each age group

Displaced midshaft fractures of the clavicle have received some attention recently, with some authors recommending operative management. It has been demonstrated that a periosteal hinge is important for fracture stability [23]. In childhood, the periosteal sleeve is thick and protects the cortex, and the bone is softer and more pliable than in adults [24]. In displaced fractures this periosteal sleeve and hinge has been mostly or completely disrupted (using Robinson's classification, displaced fractures are those that are translated by 100% or more). Sixty-six patients (35%) in our group sustained displaced fractures of the midclavicle, and all of these healed clinically with non-operative management. This included five fractures that were comminuted segmental (Robinson type 2B2), thereby having almost or complete disruption of the periosteal sleeve in at least one part of the bone. As Figure 4 shows, displaced fractures occur more commonly in children as they get older. This can be explained by the more adult type bone and periosteum as the child grows and develops. Indeed, Figures 3 and 4 demonstrate that the mean period of radiographic and clinical follow-up increased with increasing age of the children. This can in part be attributed to the larger percentage of displaced fractures being encountered with increasing age.

In our institute, radiographic evaluation of clavicle fractures is discontinued when symptoms resolve. Some authors recommend that torus/buckle fractures do not require any radiographic review whatsoever, as the incidence of complications is so small [24, 25]. In our experience, clavicle fractures in children, whether displaced or undisplaced, heal clinically, as demonstrated by the absence of pain and the return of full function. This is achieved at a mean duration of six weeks for all fractures. For all age groups, clinical followup continued for at least as long as radiographic follow-up, and in all cases radiographs were only requested when clinically indicated. All fractures healed without complication.

We would like to acknowledge certain limitations of this study. This was a retrospective study reviewing radiographs and clinical records. Clinical follow-up of patients was for a mean of 6.3 weeks. This relatively short follow-up could potentially result in late complications being overlooked. Some authors have recommended that paediatric patients with clavicle fractures require no follow-up at all [26]. This is based on the fact that most paediatric clavicle fractures heal and is justified by detailed written instructions given to parents informing them of symptoms to be aware of and when to seek further review. The vast majority of patients reviewed in this study lived locally, and all patients are advised to seek further review if they develop symptoms after discharge. Patient records were reviewed at a mean of nineteen months after injury. Had any of these patients developed late complications within this time period, this would have been documented within their records in the form of referral back to the orthopaedic clinic by their family doctor or the local emergency department. We accept that we still may have overlooked complications in the small number of patients who did not live locally or those whose symptoms were not felt severe enough to warrant further orthopaedic consultation.

Ideally, we would confirm radiographic union of all fractures. However, radiographs take time, cost money, and expose patients to radiation [27–29]. We firmly believe that radiographs should only be performed when they are likely to alter the management of the patient. Healing of paediatric clavicle fractures is known to occur within four to six weeks [30]. Our radiographic follow-up in this study was a mean of five weeks, but we do recognise that only 23% of our patients had fracture union confirmed by radiographs. Some studies have suggested that radiographs are not required at all in the assessment of clavicle fractures [31, 32], but we feel that an initial radiograph to confirm and classify the injury is appropriate even where the fracture is obvious clinically.

## 5. Conclusions

As with all fractures, clavicle fractures can develop complications regardless of management. There is evidence in the literature that highlights the aetiology and risk factors for some of these complications. Most published articles report these complications in adult patients, and there is a relative paucity of the literature available that is specifically reporting upon clavicle fractures in children. Despite this, there are a small number of reports of complications in this group. In our experience, all paediatric clavicle fractures can be treated with simple immobilisation and analgesia, without development of complications. Radiographic review of paediatric clavicle fractures is unnecessary in the absence of clinical findings suggestive of delayed union.

## Figures and Tables

**Table 1:** Robinson's classification of clavicle fractures.

Type 1A1: medial 1/5th, undisplaced, extra-articular
Type 1A2: medial 1/5th, undisplaced, intra-articular
Type 1B1: medial 1/5th, displaced, extra-articular
Type 1B2: medial 1/5th, displaced, intra-articular

Type 2A1: middle 3/5ths, undisplaced
Type 2A2: middle 3/5ths, angulated
Type 2B1: middle 3/5ths, Simple or wedge comminuted
Type 2B2: middle 3/5ths, isolated or comminuted segmental

Type 3A1: lateral 1/5th, undisplaced, extra-articular
Type 3A2: lateral 1/5th, undisplaced, intra-articular
Type 3B1: lateral 1/5th, displaced, extra-articular
Type 3B2: lateral 1/5th, displaced, intra-articular
